# The Role of Alternative Splicing in Marine–Freshwater Divergence in Threespine Stickleback

**DOI:** 10.1093/gbe/evaf105

**Published:** 2025-05-28

**Authors:** Carlos E Rodríguez-Ramírez, Catherine L Peichel

**Affiliations:** Division of Evolutionary Ecology, Institute of Ecology and Evolution, University of Bern, Bern 3012, Switzerland; Division of Evolutionary Ecology, Institute of Ecology and Evolution, University of Bern, Bern 3012, Switzerland

**Keywords:** adaptation, alternative splicing, threespine stickleback, regulatory evolution, gene expression, gill

## Abstract

Alternative splicing regulates which parts of a gene are kept in the messenger RNA and has long been appreciated as a mechanism to increase the diversity of the proteome within eukaryotic species. There is a growing body of evidence that alternative splicing might also play an important role in adaptive evolution. However, the relative contribution of differential alternative splicing (DS) to phenotypic evolution and adaptation is still unknown. In this study, we asked whether DS played a role in adaptation to divergent marine and freshwater habitats in threespine stickleback (*Gasterosteus aculeatus*). Using two published gill RNA-seq datasets, we identified differentially expressed and differentially spliced genes (DEGs and DSGs) between population pairs of marine–freshwater stickleback in the Northeast Pacific and tested whether they are preferentially found in regions of the genome involved in marine–freshwater divergence. We found over 100 DSGs, which were found more often than expected in peaks of genetic divergence and quantitative trait loci that underlie phenotypic divergence between ecotypes. DSGs and DEGs are similarly enriched in these regions. Among the different types of DS, mutually exclusive exon splicing is most strongly correlated with genetic divergence between ecotypes. Taken together, our results add support to the growing evidence that natural selection might have acted on DS and might have specifically played a role in the adaptive divergence of marine and freshwater sticklebacks. Our results also suggest that some types of DS events might contribute more than others to adaptation.

**Significance**

Alternative splicing results in the formation of different proteins from the same gene. Thus, it has recently been suggested that differences in alternative splicing could play an important role when populations or species adapt to divergent habitats. However, evidence that differentially spliced genes are under divergent selection is limited. Here, we identify genes that are differentially spliced between populations of threespine stickleback (*Gasterosteus aculeatus*) adapted to marine versus freshwater habitats. We find that differentially spliced genes are preferentially found in regions of the genome that are under divergent selection and that contribute to phenotypic differences between marine and freshwater sticklebacks, suggesting that alternative splicing contributes to divergent adaptation in this system.

## Introduction

**Fig. 1.**
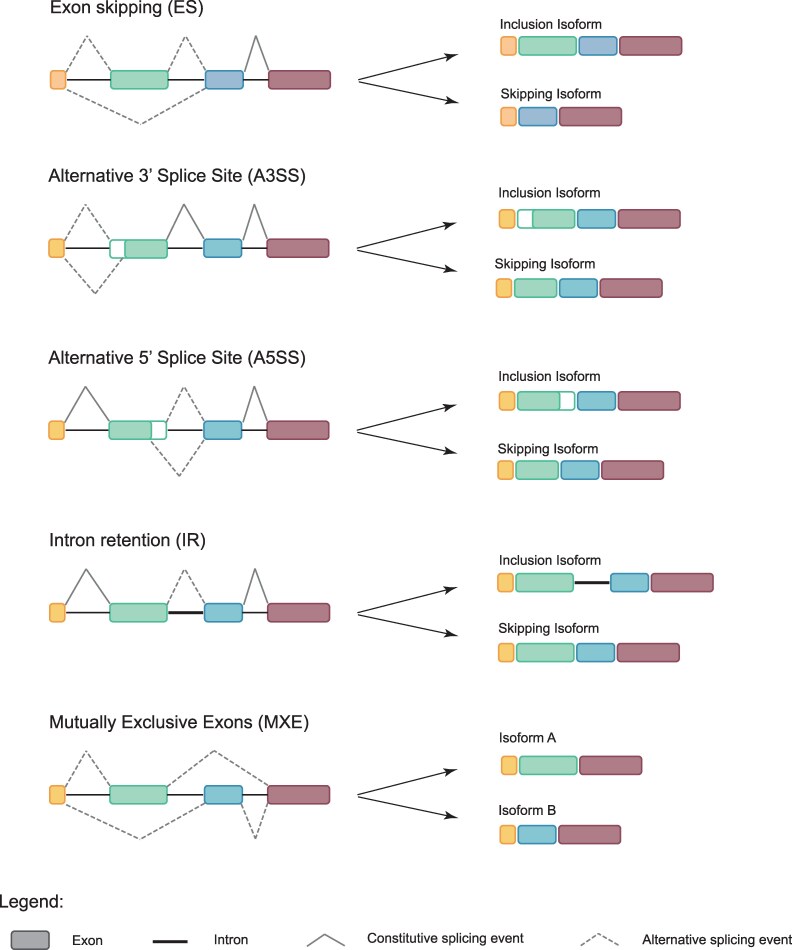
Different types of alternative splicing events. The DNA sequences are indicated to the left of the arrows, and the resulting mRNA sequences are indicated to the right. rMATs uses read pairs mapping to exon boundaries to identify alternative splicing events. Any reads mapping to the exon–exon boundaries involved in a specific splicing event or that fall in the alternative exon/intron are then classified as belonging to the “inclusion isoform” or the “skipping isoform”. After correcting for length differences between the inferred inclusion and skipping isoforms, the proportion of inclusion versus skipping reads between ecotypes is used to calculate the inclusion isoform difference metric for differential splicing (DS) between ecotypes.

Gene regulatory evolution has long been proposed to be an important driver of phenotypic evolution (King and Wilson 1975). In particular, cis-regulatory evolution has been argued to be an important driver of phenotypic evolution due to its potential to reduce antagonistic pleiotropy compared to protein evolution (Carroll 2005; Hoekstra and Coyne 2007; Stern and Orgogozo 2008; Wittkopp and Kalay 2012; Bomblies and Peichel 2022). Supporting these ideas, changes in gene expression mediated by cis-regulatory mutations have been shown to underlie phenotypic evolution and adaptation to different environments (Stern and Orgogozo 2008; Rebeiz et al. 2009; Chan et al. 2010; Wittkopp and Kalay 2012; Indjeian et al. 2016; Hill et al. 2021; Wooldridge et al. 2022). However, the literature on the role of regulatory evolution in phenotypic evolution and adaptation has for the most part focused solely on changes in gene expression and until recently has ignored other mechanisms of gene regulation, such as alternative splicing, which is common in many eukaryotic lineages (Chen et al. 2014; Bush et al. 2017; Singh and Ahi 2022; Verta and Jacobs 2022; Wright et al. 2022). When alternative splicing occurs, different combinations of exons, and sometimes introns, are included in the mature mRNA which can lead to alternative mRNA isoforms and the translation of different proteins from the same gene (Bush et al. 2017; Singh and Ahi 2022; Verta and Jacobs 2022; Wright et al. 2022). There are five types of alternative splicing (AS) events: (i) exon skipping (ES), when an alternative exon is not included in the mRNA; (ii) mutually exclusive exons (MXE), when one exon out of a group is always included in the mRNA, but never more than one of the exons at the same time; (iii) intron retention (IR), when an intron is kept in the mRNA instead of being spliced out as usual; (iv) alternative 3′ splice sites (A3SS); and (v) alternative 5′ splice sites (A5SS), when part of the 3′ or 5′ end of an exon is spliced out of the mRNA (Fig. 1). One gene can undergo a combination of these types of alternative splicing events, allowing for multiple different mRNA isoforms of a gene and leading to an overall increase in proteome diversity. This may be one reason why AS is correlated with complexity (as measured by cell-type diversity) across eukaryotic lineages (Chen et al. 2014).

The potential of AS to create different proteins has led several researchers to propose that it might play a role in phenotypic evolution and adaptation (Bush et al. 2017; Singh and Ahi 2022; Verta and Jacobs 2022; Wright et al. 2022). Indeed, recent studies have found that differential alternative splicing (for simplicity, we refer to this as differential splicing, or DS) can lead to phenotypic differences between ecotypes or species. For example, dorsal spine reduction in freshwater populations of threespine stickleback (*Gasterosteus aculeatus*) is mediated by the use of an A5SS in the first exon of *Msx2a*, a gene involved in osteoblast differentiation (Howes et al. 2017). Infrared sensation in vampire bats is linked to changes in the ratio of ES in the temperature receptor gene *Trpv1*, making it sensitive to temperatures around 30 °C instead of 40 °C (Gracheva et al. 2011). Increased lipid accumulation in two populations of the cavefish *Astyanax mexicanus* is caused by ES, resulting in a premature stop codon in the gene *per2*, a suppressor of lipid metabolism (Xiong et al. 2022). Eighteen other examples of intra or interspecific phenotypic variation mediated by alternative splicing are found in the genotype–phenotype database GePheBase (Martin and Orgogozo 2013; Courtier-Orgogozo et al. 2020), highlighting the potential of DS to cause phenotypic evolution. Several studies have identified differentially spliced genes (DSGs) between species or divergent ecotypes in human lice, cichlids, sunflowers, *Arabidopsis* and Artic charr (Tovar-Corona et al. 2015; Singh et al. 2017; Smith et al. 2018; Wang et al. 2019; Jacobs and Elmer 2021), and across environmental clines in wild house mice (Manahan and Nachman 2024). However, to our knowledge, only one study in benthic and pelagic ecotypes of Artic charr (Jacobs and Elmer 2021) has looked for evidence of divergent selection in DSGs between ecotypes of a species. Despite this growing list of examples, the relative contribution of DS to adaptation, particularly in comparison to other mechanisms of gene regulation, remains unknown.

The threespine stickleback (*G. aculeatus*) is a good model to study questions related to the genetic basis of phenotypic evolution and adaptation. This small teleost fish is distributed across the Northern Hemisphere, and the ancestral marine form independently colonized and adapted to many freshwater habitats approximately 12,000 years ago after the Last Glacial Maximum. Marine and freshwater stickleback diverge in ecology, physiology, and morphology, with the repeated evolution of many phenotypes in freshwater (Bell and Foster 1994). Genetic studies have found that several of these parallel phenotypes are due to mutations in the same gene (Colosimo et al. 2005; Miller et al. 2007; Chan et al. 2010; Ishikawa et al. 2019). Many quantitative trait loci (QTL) mapping studies have identified regions of the genome strongly associated with phenotypic divergence (Peichel and Marques 2017). In addition, global genomic studies incorporating marine and freshwater population pairs from across the Northern Hemisphere have identified parallel peaks of genetic divergence across marine–freshwater population pairs that are putatively under divergent selection. Interestingly, most of these peaks of divergence are found in noncoding regions, highlighting the important role of cis-regulatory evolution in phenotypic divergence between marine and freshwater sticklebacks (Jones et al. 2012; Verta and Jones 2019; Roberts Kingman et al. 2021).

Here, we use this system to ask whether DS could be a regulatory mechanism that contributes to adaptation to divergent environments. More precisely, we ask whether alternative splicing is important for marine–freshwater divergence in threespine stickleback. Using publicly available RNA-seq data from marine and freshwater populations from the Northeast Pacific, we ask the following questions: (i) are there DSGs between the two ecotypes?; (ii) is there evidence that these DSGs might mediate phenotypic divergence between the ecotypes?; and (iii) is there any evidence that natural selection acted on these DSGs?

## Results

### Over 100 DSGs Between Marine and Freshwater Stickleback

**Fig. 2.**
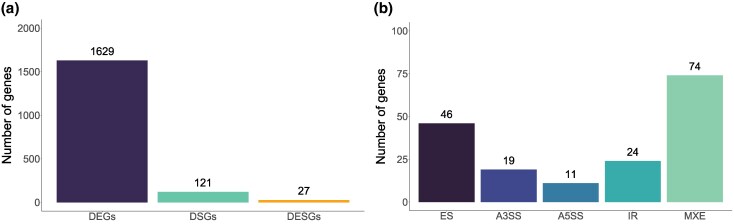
a) Number of differentially expressed genes (DEGs), differentially spliced genes (DSGs), and differentially expressed and spliced genes (DESGs) in the gill transcriptome. DEG and DSG counts in the figure do not include DESGs. b) Number of differentially spliced events of each DS type found within the 148 DSGs (including DESGs) in the gill transcriptome. Some genes have more than one type of DS.

Combining two RNA-seq datasets (Gibbons et al. 2017; Verta and Jones 2019) from gill tissue of marine and freshwater sticklebacks from Northeast Pacific populations (supplementary table S1, Supplementary Material online), we found 16,688 expressed genes (after removing mitochondrial and Y chromosome genes), of which 1,656 are differentially expressed genes (DEGs) between marine and freshwater samples. We detected alternative splicing events in 1,343 genes, of which 148 are DSGs between ecotypes (Fig. 2a, supplementary table S2, Supplementary Material online). Twenty-seven genes are simultaneously DEGs and DSGs (differentially expressed and spliced genes, or DESGs). Not all DEGs and DSGs identified in the merged dataset are significant when the two datasets are analyzed individually. However, there is a positive correlation in fold-change and isoform differences for all DEGs and DSGs between the individual and merged datasets (supplementary fig. S1, Supplementary Material online). We found all five types of differential splicing events among the DSGs, with some genes having more than one type (Fig. 2b). Differential MXE is the most common and is present in 74 DSGs; this is followed by 46 DSGs with differential ES, 24 DSGs with IR, 19 DSGs with A3SS, and 11 DSGs with differential A5SS (Fig. 2b, supplementary table S2, Supplementary Material online). There is no enrichment of GO Terms in DEGs or DSGs (supplementary table S3, Supplementary Material online).

### DEGs and DSGs Are Significantly Enriched in Some Categories of QTL

**Fig. 3.**
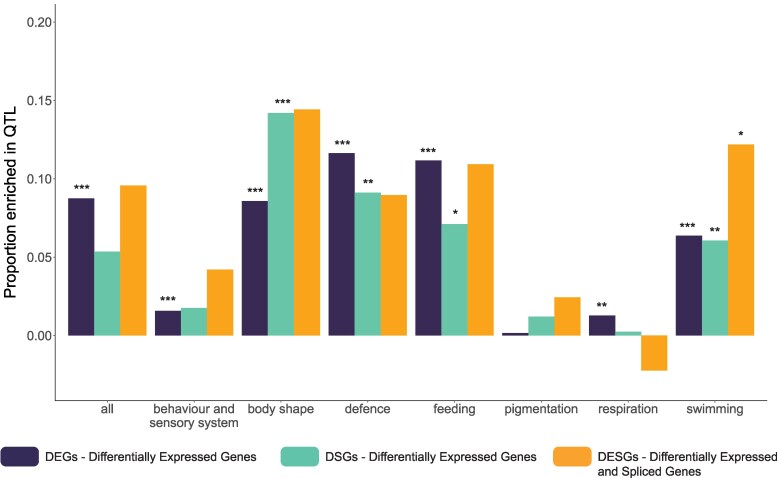
QTL enrichment analysis results for DEGs, DSGs, and DESGs. Bars represent the proportion of DEGs, DSGs, or DESGs in a QTL for a given category minus the proportion of background genes in a QTL for that category. Asterisks represent significance levels for the QTL enrichment test (permutation test, 1,000 permutations): *P < 0.05; **P < 0.01; ***P < 0.001.

To test whether DEGs and DSGs might be mediating phenotypic divergence between marine and freshwater sticklebacks, we tested their enrichment in 316 QTL that underlie traits that diverge between Pacific marine and freshwater populations (supplementary table S4, Supplementary Material online) (Peichel and Marques 2017; Liu et al. 2022; Rennison and Peichel 2022). The QTL span most of the genes in the gill transcriptome: Out of the 16,688 genes expressed in our transcriptome data, 12,124 genes (72.8%) are inside at least one QTL. We found that DEGs are enriched in the overall set of QTL, as well as in most phenotypic sub-categories (Fig. 3, supplementary table S5, Supplementary Material online). Meanwhile, the DSGs are not enriched in the overall set of QTL, but they are enriched in QTL sub-categories associated with body shape (*P* < 0.001), defense (*P* = 0.007), feeding (*P* = 0.044), and swimming (*P* = 0.006) (permutation tests, 1,000 permutations). The set of 27 DESGs are only enriched in QTL associated with swimming (*P* = 0.023; permutation tests, 1,000 permutations) (supplementary table S5, Supplementary Material online). However, DESGs have a tendency to be enriched in most QTL categories (Fig. 3), so the lack of significant enrichment in QTL might be due to a lack of statistical power with such a small set of genes.

### DEGs and DSGs Are Significantly Enriched in EcoPeaks

**Fig. 4.**
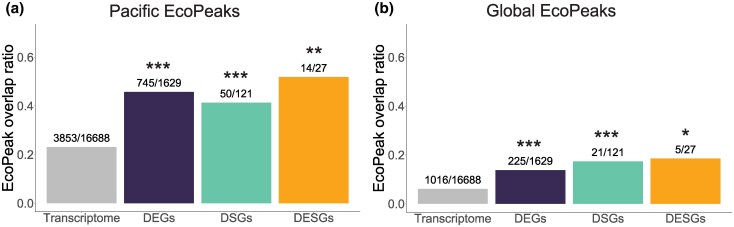
Proportion of DEGs, DSGs, DESGs, and transcriptome genes overlapping a) Pacific and b) Global EcoPeaks. Transcriptome genes are all genes that passed the minimum expression filter (i.e. the background of the DEGs). DSGs and DESGs were compared to their respective backgrounds, but for simplicity they are not represented in the figure (supplementary table S7, Supplementary Material online). The number of genes of each category inside the EcoPeaks relative to the total number of genes in that category is shown above each bar. Asterisks represent significance levels for the EcoPeak enrichment test (permutation test, 1,000 permutations): *P < 0.05; **P < 0.01; ***P < 0.001; NS—not significant, *P* > 0.05.

To determine whether DEGs and DSGs might be under divergent selection, we tested whether they are significantly enriched in EcoPeaks, which are regions of the genome with peaks of genetic divergence between multiple marine and freshwater populations from either the Northeast Pacific (“Pacific EcoPeaks”) or from the Northeast Pacific, California, and Europe (“Global EcoPeaks”) (Roberts Kingman et al. 2021). The Pacific EcoPeaks range in size from 17 bp to 5.3 Mbp; each EcoPeak includes from 0 to 199 of the genes in the transcriptome. Together, the Pacific EcoPeaks include 23.1% of the genes in our transcriptome (3,853 out of 16,688) (supplementary tables S2 and S6, Supplementary Material online). We found that 45.7% of DEGs (745 out of 1629), 41.3% of DSGs (50 out of 121), and 51.9% of DESGs (14 out of 27) are inside the Pacific EcoPeaks. This is significantly more than the 23.1% to 23.7% of background genes that are in these regions of the genome (DEGs and DSGs: *P* < 0.001; DESGs: *P* = 0.002; permutation tests, 1,000 permutations) (Fig. 4a, supplementary table S7, Supplementary Material online). DEGs and DSGs are similarly enriched in Global EcoPeaks. We found 13.8% of DEGs (225 out of 1629), 17.3% of DSGs (21 out of 121), and 18.5% of DESGs (5 out of 27) inside Global EcoPeaks. This is significantly more than the 6.1% to 6.9% of background genes that are in these regions of the genomes (DEGs and DSGs: *P* < 0.001; DESGs: *P* = 0.029; permutation tests, 1,000 permutations) (Fig. 4b, supplementary table S7, Supplementary Material online).

Since EcoPeaks are regions of high genetic divergence between ecotypes, DEGs and DSGs within them are the most likely to result from divergent cis-regulatory mutations. Likewise, since QTL are regions of the genome associated with phenotypic divergence between the ecotypes, DEGs and DSGs within them are the most likely to be playing a role in phenotypic differences between ecotypes. Thus, we hypothesized that DEGs and DSGs that are simultaneously found within an EcoPeak and a QTL are the strongest candidates to be playing an adaptive role in marine–freshwater divergence. Focusing on DSGs, we found 24 such genes, eight of which are uncharacterized (supplementary tables S2 and S8, Supplementary Material online). For the remaining 16 genes, we asked whether DEGs and DSGs overlapping both Pacific EcoPeaks and QTL are enriched in any particular biological functions. However, we found that these genes are not significantly enriched in any GO Term category (supplementary table S3, Supplementary Material online). Thus, we used the GeneCards database to manually investigate the function of the DSGs found within both Global EcoPeaks and QTL. We found multiple genes involved in essential amino acid metabolism, chromatin remodeling, immunity, vesicle transport, and muscle function (supplementary table S8, Supplementary Material online).

Zooming into the types of differential splicing events underlying the DSGs, we found that DSGs with significant MXE and ES events are enriched in Pacific EcoPeaks (56.8% and 37.0%, respectively; *P*-values of <0.001 and 0.025; permutation test, 1,000 permutations) (supplementary fig. S2a and table S7, Supplementary Material online). Though not significant, DSGs with A3SS show a similar trend, with 7 out of 19 (36.8%) found in EcoPeaks (*P* = 0.258; permutation test, 1,000 permutations). DSGs with MXE, ES, and IR are also significantly enriched in the Global EcoPeaks (supplementary fig. S2b and table S7, Supplementary Material online).

**Fig. 5.**
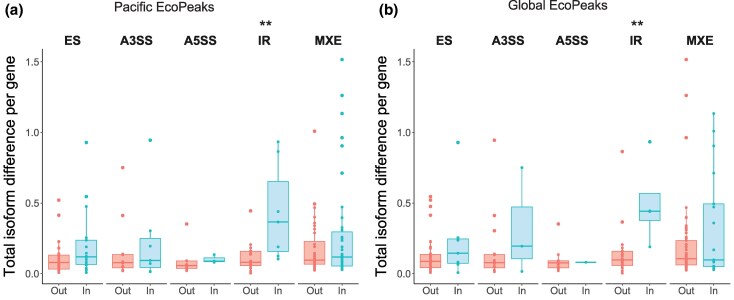
Comparison of the isoform difference between DS events outside (Out) and inside (In) of a) Pacific and b) Global EcoPeaks, divided by type of DS event. Asterisks represent significance levels for a permutation test on the median difference of the isoform distributions of DS events inside and outside of EcoPeaks (permutation test, 1,000 permutations): **P < 0.01.

Finally, we examined whether there is a difference in the overall fold-change in expression (DEGs) or the inclusion isoform change (“isoform difference” in DSGs) between genes inside versus outside of Pacific and Global EcoPeaks. We found no difference in the expression fold-change between DEGs inside and outside of either type of EcoPeaks (supplementary fig. S3 and table S9, Supplementary Material online). However, we found that DSGs had a slightly higher average isoform difference when inside of Global EcoPeaks (supplementary fig. S3 and table S9, Supplementary Material online). We further explored this result by discriminating between types of DS events within the DSGs. This analysis revealed that IR DS events within both Pacific and Global EcoPeaks had a larger average isoform difference per gene than those outside EcoPeaks (Fig. 5, supplementary table S9, Supplementary Material online).

### No Strong Correlation Between Transcriptome-Wide Genetic Divergence and Differential Expression and Splicing

To determine to what extent marine–freshwater splicing divergence is correlated with marine–freshwater genetic divergence, we used the SNP-level genetic distance measurements from the EcoPeak data (Roberts Kingman et al. 2021). Briefly, these were *P*-values resulting from a Fisher Exact Test for the probability of an imbalance in allele counts between ecotypes at each SNP. A *P*-value of <0.05 means that the SNP differs in allele frequencies between ecotypes and suggests divergent selection. We averaged the *P*-values for all SNPs within a gene and used this average SNP *P*-value as a proxy for genetic distance between the ecotypes at that gene. We found a very weak positive correlation between the total isoform difference of all DS events in the gene and genetic distance (*R*^2^ = 0.014, *P* = 5.87e^−06^) (supplementary fig. S4b, Supplementary Material online). We found a similarly weak positive correlation between genetic distance and the expression fold-change between ecotypes (*R*^2^ = 0.018, *P* = 2.2e^−16^) (supplementary fig. S4a, Supplementary Material online). These weak correlations are likely driven by the EcoPeaks. When we separated the data inside and outside of the EcoPeaks, the correlations between genetic distance and isoform difference/fold-change are weaker and no longer significant in the case of total isoform difference (supplementary fig. S4c to f, Supplementary Material online).

### Comparisons of Isoform Difference and Genetic Divergence Among Types of DS Events

**Fig. 6.**
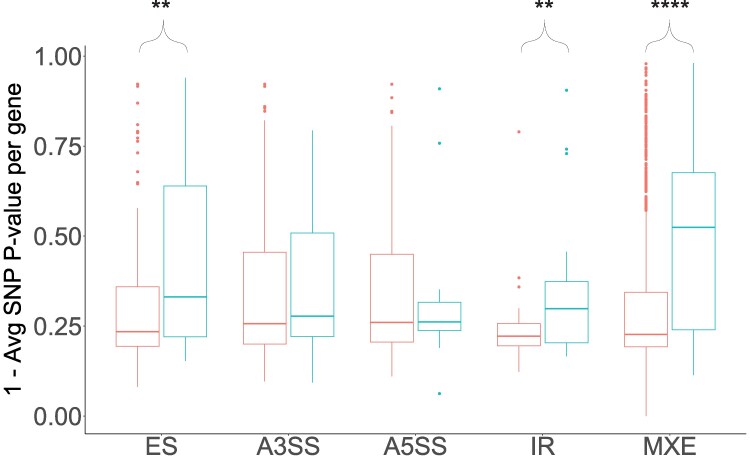
Distributions of average SNP *P*-value for significant DSGs (blue) and non-DSGs (red) per splicing type; non-DSGs are genes that are alternatively spliced but not significantly differentially spliced between marine and freshwater samples. Higher values on the *y* axis mean that the SNPs within the genes have on average a more significant genetic imbalance between ecotypes as tested by a Fisher Exact Test (Roberts Kingman et al. 2021). To make the *y* axis more intuitive, the average SNP *P*-value is subtracted from 1 so that larger values represent greater genetic divergence. Asterisks indicate whether DSGs have a significantly higher genetic divergence median than non-DSGs (permutation test, 10,000 permutations): **P < 0.01; ****P < 0.0001.

To gain insights into whether different types of splicing events could be more important to adaptation than others, we compared the genetic divergence and isoform difference among the five types of DS events. We found no differences in total isoform difference (the sum of the isoform difference of all DS events of the same type) among the five types of DS events (supplementary fig. S5a and table S10a, Supplementary Material online). However, we found that DSGs in which MXE is the DS event with the highest isoform difference tend to have a higher genetic divergence than DSGs in which the highest isoform difference is from another type of DS event. However, this tendency was only significant when comparing MXE to IR (supplementary fig. S5b and table S10b, Supplementary Material online). However, the small number of DSGs, particularly when subset among the five types of DS events, gives little statistical power to this analysis. Thus, we also compared the genetic divergence of DSGs to their non-DSG counterparts for each type of DS event. We find that DSGs with differentially spliced MXE, ES, and IR between ecotypes have a significantly higher genetic divergence than genes that have alternative MXE, ES, and IR, respectively, but that do not differ between ecotypes (Fig. 6, supplementary table S11, Supplementary Material online). This suggests that DSGs with these types of splicing are more likely to have been targeted by selection between marine and freshwater sticklebacks.

## Discussion

The magnitude of the role of alternative splicing in adaptation is still unknown. We sought to tackle this question by assessing the role of alternative splicing in the marine–freshwater divergence of threespine stickleback (*G. aculeatus*). We show that there are more than 100 DSGs in the gill transcriptome between marine and freshwater stickleback populations from the Northeast Pacific. DSGs are enriched not only in regions under putative divergent selection in the genome, but also within QTL underlying phenotypic divergence between ecotypes in the Pacific. Furthermore, the enrichment of DSGs in these regions of the genome was similar to the enrichment found for DEGs, suggesting that DSGs might be as important for adaptation as DEGs. Among the five types of DS, differential MXE events are the most commonly divergent between ecotypes and are more genetically divergent than non-DSGs with MXE. This suggests that MXE might be the type of alternative splicing that has contributed the most to the marine–freshwater adaptive divergence in threespine stickleback. Taken together, our results suggest that alternative splicing might play a role in marine–freshwater divergence in threespine stickleback.

### Over 100 Genes Have Changes in Alternative Splicing Between Marine and Freshwater Sticklebacks in Gill

To determine the role of alternative splicing in the freshwater–marine divergence in threespine stickleback, we first assessed the extent of differential expression and differential splicing between ecotypes. We found 1,629 DEGs, 121 DSGs, and 27 DESGs (Fig. 2a). This disparity in abundance of DEGs and DSGs is a pattern commonly found in other studies (Grantham and Brisson 2018; Jacobs and Elmer 2021; Steward et al. 2022; Rodríguez-Ramírez et al. 2023; Manahan and Nachman 2024), though not always (Singh et al. 2017). This might suggest that differential splicing is less commonly used than differential expression for adaptation. Since AS directly affects the protein sequence, it is possibly under stronger purifying selection than differential expression. However, it is important to note that methods to detect AS in short-read data have greater limitations in their ability to identify and quantify changes in splicing than methods for identification of DEGs in short-read data. For example, methods based on splice junctions like the one used in this study and others (e.g. Steward et al. 2022) work mainly with reads overlapping these junctions. Because most reads cannot be used for analyses, the power of the DS analyses is greatly reduced compared to the DE analyses, which can use most reads in the transcriptome. Methods based on differential exon usage have been used in other studies (e.g. Singh et al. 2017; Jacobs and Elmer 2021; Rodríguez-Ramírez et al. 2023) and can use most of the same reads as DE analyses but cannot detect certain types of splicing (i.e. A3SS and A5SS). Thus, all DS studies based on short-read RNA-seq data are likely to underestimate the extent of DS. In contrast, long-read RNA sequencing methods like Iso-seq provide information on the full mRNA sequence and have revealed many previously unknown isoforms both in animals and plants (Singh and Ahi 2022). Future studies using long-read RNA sequencing technologies are essential to properly assess the relative role of DEGs and DSGs in adaptation.

Another caveat of our analyses is that one of the RNA-seq datasets used was taken from wild-caught individuals acclimated to different salinities in the laboratory (Gibbons et al. 2017). Thus, the DEGs and DSGs we identified in this dataset could result from both genetic and plastic effects. Nonetheless, our conclusions are based on DEGs and DSGs that were identified in the merged analyses of both datasets. Despite the many biological and technical differences between the two datasets (supplementary table S1, Supplementary Material online), there is a strong correlation between the log fold-change in expression and isoform differences between the two datasets for genes that were identified in the merged analyses (supplementary fig. S1, Supplementary Material online). Thus, the overall conclusions should not be biased by unequal contributions from the two datasets. Furthermore, as detailed below, we find evidence that these DEGs and DSGs are enriched in QTL that underlie phenotypic differences between marine and freshwater sticklebacks, as well as genomic regions under divergent selection between marine and freshwater sticklebacks. Thus, our analyses have likely captured differences in expression and splicing that were due to genetic differences between the marine and freshwater populations used in this dataset (Gibbons et al. 2017).

### DSGs Are Associated With Regions Underlying Phenotypic Divergence in Body Shape, Defense, and Feeding Between Marine and Freshwater Stickleback

Consistent with a role for DEGs and DSGs in mediating phenotypic divergence between marine and freshwater sticklebacks, we found that DEGs and DSGs were enriched in QTL associated with a variety of phenotypes. While DEGs were enriched in more QTL, DSGs were still associated with QTL underlying phenotypes like body shape, defense traits and feeding traits. However, an important caveat is that these QTL are mostly associated with phenotypes completely unrelated to gill tissue, except for some of the feeding trait QTL (supplementary table S4, Supplementary Material online). The expression of genes associated with these QTL in gill tissue could be due to several non-mutually exclusive reasons. One possibility is that some of these genes have pleiotropic effects in both gill and the tissues associated with the QTL. For example, the gene *stat3* is among the most divergent DSGs in the dataset (supplementary table S8, Supplementary Material online) and is a pleiotropic transcription factor with described roles in multiple biological processes including skeletal development, hair maintenance, immunity, and cellular respiration (Levy and Lee 2002; O'Shea et al. 2002; Wegrzyn et al. 2009; Hillmer et al. 2016; Zhou et al. 2021). Furthermore, *stat3* in mice is known to produce two alternative splicing isoforms: a full-length isoform, *stat3α*; and a truncated isoform, *stat3β.* The isoforms have different phenotypic effects and are thought to partially explain the high pleiotropy of this gene (Maritano et al. 2004). A second possibility is that most of the QTL have very low resolution and span large regions of the genome, so most of the genes found within them are unlikely to be related to the focal QTL phenotype. An enrichment of DEGs and DSGs in these QTL could occur if these genes affect other unmapped phenotypes that are linked to these QTL, consistent with the QTL clustering observed in stickleback (Peichel and Marques 2017). A final possibility is that strong selection for DE and DS in a gene within a specific tissue could allow the fixation of regulatory variants that cause leaky expression and splicing in unrelated tissues like gill. Mutations in cis-regulatory elements are known to affect gene expression noise (Richard and Yvert 2014), and the strength of selection against noise depends on the function of the gene and its position in the gene pathway (Barroso et al. 2018). Thus, it is possible that when rapid adaptation to different environments occurs, regulatory variants that are favorable in one tissue might get fixed despite increasing the transcriptional noise of that gene in other tissues. Indeed, many of the DEGs and DSGs have relatively small changes in expression or splicing (supplementary table S2, Supplementary Material online), which is what we would expect if some of these genes represented some sort of transcriptional leakage resulting from divergent selection between marine and freshwater stickleback in other tissues. However, given that many adaptive alleles in stickleback are more than 1 million years old (Colosimo et al. 2005; Roberts Kingman et al. 2021), we would expect that any alleles with such negative pleiotropic effects might be rare. Long-term studies to identify the causative genes and alleles in these QTL will clarify the role of these DSGs in phenotypic divergence between marine and freshwater sticklebacks.

### DSGs Are Associated With Regions Under Divergent Selection Between Ecotypes

Having determined that DSGs are enriched in regions of the genome associated with phenotypic divergence between marine and freshwater sticklebacks, next we asked whether the same was true for the smaller subset of the genome with putative signatures of divergent selection between ecotypes. We used the EcoPeaks database (Roberts Kingman et al. 2021) and found that DEGs and DSGs were enriched in both Pacific and Global EcoPeaks (Fig. 4). This suggests that DSGs are as likely as DEGs to be under divergent selection between threespine stickleback ecotypes in Northeast Pacific populations. It is possible that hitchhiking with the actual targets of selection within the EcoPeaks led to elevated levels of neutral genetic divergence in these regions, resulting in cis-regulatory changes that lead to DE and DS. However, we find only a very weak linear correlation between genetic divergence within the EcoPeaks and the magnitude of DE and DG across the gill transcriptome (supplementary fig. S4, Supplementary Material online), suggesting that the DEGs and DSGs inside of the Pacific EcoPeaks are not just a side-effect of local genetic divergence. However, it is important to note that we do not have genomic sequencing data for all the individuals used to identify DEGs and DSGs, so we cannot directly look at the correlation between genetic distance and DE and DS. In the future, it would be particularly interesting to generate transcriptomic and genomic data from the same individuals and to then correlate genetic divergence within putative cis-regulatory elements with the extent of DE and DS between marine and freshwater sticklebacks.

### MXE, IR, and ES May Play an Important Role in Marine–Freshwater Divergence

Alternative splicing can generate different types of splicing events (Fig. 1). To test whether they might play different roles in adaptation, we assessed the presence of five types of DS events in our data. While we found all five types of DS events in the DSGs of our dataset, MXE, ES, and IR events were more common, and they were also significantly enriched in EcoPeaks (supplementary fig. S2, Supplementary Material online). Furthermore, we also find that these three types of DS events have a higher genetic divergence their non-DSGs counterparts (Fig. 6). Finally, we find that differentially spliced IR events have higher average isoform differences per gene in EcoPeaks than outside of EcoPeaks, suggesting selection for higher splicing divergence between ecotypes in these types of splicing events. Taken together, these results suggest that MXE, ES, and IR are more likely than A3SS and A5SS to be under selection between the marine–freshwater sticklebacks and play a role in their adaptive divergence.

It is not clear why A3SS and A5SS would be less used in adaptation. They are the least common types of DS events in our dataset and did not stand out in any of our analyses. It is possible that this is partially due to a weaker statistical power in the analyses due to their smaller sample size. However, IR was not much more common than A3SS, and it was still significant in many analyses. Thus, if A3SS and A5SS had a strong effect in any of these analyses, we would probably have detected it. Just like ES and IR, A3SS and A5SS remove and add mRNA sequence and thus have the potential to increase proteomic diversity. However, most of the time, this likely leads to aberrant proteins, truncated proteins or degradation of the mRNA by nonsense-mediated mRNA decay (NMD). Nonetheless, all of these can still be a mechanism for indirect down-regulation of genes, which could be adaptive. This is the case for the gene *Msx2a*, which underlies dorsal spine differences between marine and freshwater threespine sticklebacks. The freshwater allele of *Msx2a* has increased A5SS splicing of the first exon of this gene, which introduces an early stop codon and leads to a truncated nonfunctional version of the protein, leading to shorter dorsal spines (Howes et al. 2017). Similarly, an ES event in the gene *TBXT* removes part of the transcriptional regulation domain of the protein and leads to the loss of tail in apes (Xia et al. 2024). Overall, ES and IR probably tend to add or remove larger sequences to the mRNA than A3SS and A5SS, which could increase the probability of new protein domains to emerge. This might also make down-regulation of genes by NMD more reliable through ES and IR than A3SS and A5SS. Indeed, IR has been found to both regulate gene expression by NMD and to contribute to functional isoforms (Wong et al. 2016). ES has similarly been found to regulate the incorporation of “poison” exons in mammalian splicing regulator (SR family) genes that lead to NMD but are important for cell fitness (Wright et al. 2022).

Furthermore, ES can also regulate new exons that emerge in intronic sequences. This can occur for example, through exonization of intronic transposable elements (TEs) (Lev-Maor et al. 2007; Sorek 2007; Wright et al. 2022). Studies in human and mice suggest that most new exons are alternatively spliced and expressed at low frequencies, making them nearly neutral (Xing and Lee 2006; Sorek 2007). This can allow new exons to evolve and potentially acquire a new function over time. For example, primates possess many lineage-specific exons that have originated from a class of TEs known as *Alu* elements. While their function is still not clear, *Alu*-containing exons can be expressed. Although they tend to be alternatively skipped (Sorek et al. 2002), some have been found to contribute to the sequence of proteins (Lin et al. 2016; Martinez-Gomez et al. 2020; Wright et al. 2022). While over 79% of new exons in humans are predicted to be deleterious (Sorek 2007), some are known to have acquired new functions. For example, the human ADAR2 gene is an RNA editing enzyme with a primate-specific exon 8 derived from an *Alu* element. This exon can be included in the catalytic region of the protein, which still works with the same substrate but with an altered catalytic activity (Sorek 2007).

Despite the potential of ES and IR to contribute to adaptation, MXE is the type of splicing that stood out the most in our data. This is the most common DS event (Fig. 2), the most enriched in EcoPeaks (supplementary fig. S2, Supplementary Material online), and the DS event found in the DSGs with the greatest isoform differences and genetic divergence (Fig. 6; supplementary fig. S5, Supplementary Material online). MXE is a type of splicing that switches one alternative exon for another in the mRNA (Fig. 1). This allows for modular changes in the protein structure where one protein domain can be switched for a different one without affecting the integrity of the protein. One extreme example of this is the *Dscam* gene in *Drosophila melanogaster*, which encodes for a transmembrane protein that is important for neuronal connection and self-avoidance. The gene has 115 exons, of which 95 belong to one of four clusters of mutually exclusive exons and has been found to produce up to 18,496 isoforms (Sun et al. 2013), more than the number of genes in the genome of *D. melanogaster*. Furthermore, MXE is thought to be associated with exon duplications (Kondrashov 2001; Letunic 2002; Wright et al. 2022). Similar to gene duplications and exonization of intronic sequences, MXE can allow for the evolution of new functions in one of the paralogous exons while maintaining its ancestral function in the other paralog. Evolving new exons through duplications rather than exonization of TEs could be advantageous in that duplicated exons are immediately able to code for a functioning part of the existing protein. Thus, exon duplication and combined with MXE could promote the evolution of alternative protein domains that might become advantageous when the species adapts to a different environment. Taken together, our results suggest that MXE could be a powerful mechanism to maintain standing genetic variation in protein isoforms and could play an important role in the divergence of marine and freshwater stickleback in the Pacific.

## Conclusion

The role of alternative splicing in adaptation is still poorly understood. However, alternative splicing can be quite a versatile regulatory mechanism that can both indirectly down-regulate the expression of a gene by creating nonfunctional isoforms and mediate modular changes in the protein by permuting different exons from the gene into the mRNA. Consistent with a growing body of evidence in other systems, we find evidence for a role of alternative splicing in the marine–freshwater adaptive divergence of threespine stickleback. DSGs between marine and freshwater populations are enriched in QTL underlying phenotypic divergence between ecotypes, and DSGs are as enriched as DEGs in regions of the genome putatively under divergent selection between marine and freshwater populations. We also find evidence that different types of alternative splicing might contribute differently to adaptation, with MXE standing out in our data and suggesting that the modular change of exons could be particularly important for adaptation. Finally, our results are quite likely an underestimate of the true extent of differential splicing between marine and freshwater sticklebacks. Limitations of geographical representation, number of tissues, developmental time points, and short-read data mean that we are probably detecting only a small part of the alternative splicing events in the threespine stickleback transcriptome. Future studies with more populations and tissues, long-read sequencing, and functional analyses will be essential to have a more precise picture of the role of alternative splicing in marine–freshwater divergence in threespine stickleback.

## Materials and Methods

### RNA-Seq Data

We searched the NCBI Sequence Read Archive (SRA) for publicly available RNA-seq data that met three criteria: (i) the data included samples from marine and freshwater population pairs of threespine stickleback (*G. aculeatus*); (ii) the data came from the same tissue, so it could be merged and compared; and (iii) the data came from Pacific populations, since these are the ones with the most complete QTL and genetic divergence data (Peichel and Marques 2017; Roberts Kingman et al. 2021). Following these criteria, we found data from two RNA-seq studies in stickleback gill tissue (Gibbons et al. 2017; Verta and Jones 2019) (supplementary table S1, Supplementary Material online). The Verta and Jones (2019) data is from first-generation descendants of wild-caught individuals grown in a common garden in the lab at 3.5 parts per thousand (ppt) salinity. From this dataset, we used four marine and four freshwater individuals from the Little Campbell River (British Columbia, Canada). The Gibbons et al. (2017) data included ten marine wild-caught individuals from Oyster Lagoon (British Columbia, Canada) that spent four weeks at 20 ppt in the laboratory before being gradually moved to either 0.0 ppt (five individuals) or 30 ppt (five individuals) for three months, and ten freshwater wild-caught individuals from Trout Lake (British Columbia, Canada) that spent four weeks at 2.0 ppt in the laboratory before gradually being exposed to either 0.0 ppt (five individuals) or 30 ppt (five individuals) for three months.

### RNA-Seq Data Preprocessing

Quality control of the RNA-seq read libraries was done with FastQC v0.11.7 (https://www.bioinformatics.babraham.ac.uk/projects/fastqc/). Reads were quality trimmed using Trimmomatic v0.36 (Bolger et al. 2014). Reads where both paired ends passed quality filtering were then mapped against version 5 of the *G. aculeatus* genome (Nath et al. 2021) using STAR v2.7.10b (settings: --twopassMode --chimSegmentMin [1/3 of read length]--alignSJDBoverhangMin 3 --alignIntronMin 70 --alignIntronMax 562000 --alignMatesGapMax 562000 --limitSjdbInsertNsj 2000000). Library quality metrics from FastQC and alignment quality metrics from STAR and featureCounts were summarized and visualized with MultiQC v1.14 (Ewels et al. 2016) to assess sample quality (supplementary table S1, Supplementary Material online).

### Sexing of Samples

Since samples from Gibbons study (Gibbons et al. 2017) had no sex information, their sex was inferred based on gene expression clustering with the sexed samples from the Verta and Jones study (Verta and Jones 2019). We used the multidimensional scaling (MDS) analysis implemented in the *plotMDS()* function of the *limma v3.40.6* R package (Ritchie et al. 2015). This analysis clusters samples based on the 500 genes with the highest gene expression differences between each pair of samples. In this analysis, we used our gene expression count data after filtering lowly-expressed genes but before removing the Y and mitochondrial chromosomes (see following section). We found that expression of genes on chromosome Y strongly separated male and female samples from Verta and Jones (2019), with samples from Gibbons et al. (2017) strongly mirroring this clustering. Based on whether they were clustering with male or female samples on the MDS plot of the Y chromosome genes, samples from Gibbons et al. (2017) were inferred to be male or female, respectively. However, outside of chromosome Y, we found no strong sex-biased clustering in the gill transcriptomes (supplementary fig. S6, Supplementary Material online).

### Identification of DEGs

We obtained count tables of the RNA-seq reads mapped to each gene in the genome using the gene annotations from NCBI (build 100) for version 5 of the *G. aculeatus* genome (https://www.ncbi.nlm.nih.gov/datasets/taxonomy/481459/) and *featureCounts* from the *Subread* v2.0.3 (Liao et al. 2014) package. We excluded reads where one of the pairs was unaligned, or when the two pairs mapped to different chromosomes or different strands. Next, we used the *edgeR* v3.28.1 package (Chen et al. 2008) in *R* v3.6.1 (R Core Team 2019) for the differential expression analysis. First, we filtered lowly-expressed genes using the *filterByExpr()* function, which removes genes with less than ten counts in a minimum number of samples based on sample size (for further details, consult the *filterByExpr()* documentation). For our data, this meant that genes with less than ten counts in nine or more samples were removed. We also removed genes from the Y and mitochondrial chromosomes since these were not present in either the EcoPeak or QTL data used in downstream analyses (more details in EcoPeak and QTL Enrichment Analyses). Second, we fit the count data in *edgeR* to a negative binomial general linear model to control for batch effects present in the datasets. We used a ∼(study + sex + ecotype) model to control for the effect of having data from two different studies and for sex-specific differences in expression. Note that it was not possible to include salinity treatment as a factor in the model because different salinity treatments were only used in the Gibbons et al. (2017) dataset (but see below). Third, we ran a quasi-likelihood F test on the fitted data to test for differential expression between marine and freshwater samples.

To assess the effect of combining data from two different studies, DEGs were also similarly identified for the two datasets separately, using a model without the “study” factor. We also looked at the correlation of gene expression fold-change between the two individual datasets for DEGs in the merged dataset using a nonparametric linear model based on ranks implemented in the *R* package *Rfit* v0.24.2 (Kloke and McKean 2012).

Finally, we tested the effect of the salinity treatment in the Gibbons et al. (2017) dataset on the gene expression analysis. This “treatment” effect could not be explicitly included in the model above because the Verta and Jones (2019) dataset does not include a salinity treatment. Nonetheless, we examined the effect of adding “treatment” as a fixed effect in the gene expression analyses of the Gibbons dataset. First, we performed an MDS analysis (as described in the Sexing of Samples section) and found no clustering by “treatment” in the first two dimensions, which are instead driven by sex in the first dimension and ecotype in the second dimension (supplementary fig. S7a, Supplementary Material online). Second, we compared plots of the Biological Coefficient of Variation (BCV) of the Gibbons dataset with and without “treatment” in the model using the *plotBCV()* function from EdgeR; the model with the “treatment” effect fits the data better, but only slightly (supplementary fig. S7b and c, Supplementary Material online). Finally, we compared the overlap of DEGs identified in the Gibbons dataset using a model with and without “treatment” effect, as well as the overlap with the DEGs identified in the main merged dataset. While adding the “treatment” effect did increase the power to identify DEGs in the Gibbons dataset, the vast majority of the DEGs we identified without the “treatment” effect are a subset of those found with the “treatment” effect (supplementary fig. S7d, Supplementary Material online). Together, these analyses suggest that not including this “treatment” effect in the analyses mostly just increases the noise and reduces power, rather than biasing the set of DEGs used for analyses.

### Identification of DSGs

To identify DSGs, we used a method based on the identification of splicing events in RNA-seq data implemented in the program *rMATs* v4.1.2 (Shen et al. 2014). First, rMATs uses reads that STAR maps to exon boundaries in the mRNA or read pairs that map to different exons in a gene, to identify five types of alternative splicing events: exon skipping (ES), alternative 5′ and 3′ start sites (A5SS and A3SS), intron retention (IR), and mutually exclusive exons (MXE). For each splicing event, rMATs defines an inclusion isoform and a skipping isoform (Fig. 1), counts how many reads map to each isoform, and calculates an Isoform Inclusion Difference metric (Shen et al. 2014), also known as Percent Spliced-In, or PSI (Grantham and Brisson 2018; Rogers et al. 2021). This metric, which we will refer to as isoform difference, measures how much the ratio of the inclusion isoform changes between treatments (Shen et al. 2014). Since rMATs does not include batch-correction or minimum expression filters, we took the raw counts of the splicing events identified by *rMATs* and used the *edgeR* function *filterByExp()*, to filter out lowly-expressed splicing events. Next, in a two-step process, we batch-corrected the counts first for the study effect in the data, and then for the sex of the samples using the function *ComBat-seq()*, from the package *sva* v3.35.2 (Zhang et al. 2020). Finally, using the “*—task stat*” mode in *rMATs*, we re-calculated the ecotypic isoform differences of each splicing event using the batch-corrected counts and performed the rMATs statistical test for differential splicing, using default settings. Genes with at least one significant differentially spliced event (DS event) between ecotypes were categorized as DSGs.

To assess the effect of combining data from two different studies, DS events were also similarly identified for the two datasets separately but only using *ComBat-seq()* to correct the counts for sex. We also looked at the correlation of isoform differences between the two individual datasets for DS events in the merged dataset using a nonparametric linear model based on ranks implemented in the *R* package *Rfit* v0.24.2 (Kloke and McKean 2012).

### EcoPeak and QTL Enrichment Analyses

To test whether DEGs and DSGs might be under divergent selection, we asked whether they were enriched in “EcoPeaks”, regions of the genome with peaks of genetic divergence between multiple marine and freshwater populations from across the Northern Hemisphere (Roberts Kingman et al. 2021). The dataset is divided into Northeast Pacific (which we will refer to as Pacific for simplicity) and Global EcoPeaks depending on the samples used. The Pacific EcoPeaks were identified by comparing 12 marine and 57 freshwater populations from Alaska (US), Haida Gwaii (Canada), British Columbia (Canada), and Washington State (US). The Global EcoPeaks were identified by comparing 28 marine and 56 freshwater populations from the Northeast Pacific, California, and Europe. EcoPeaks were identified using two approaches: (i) a window-based genetic distance approach; and (ii) a SNP-level statistical test for imbalance of genetic variants between marine and freshwater populations. Sensitive EcoPeaks were defined when an FDR of 5% was obtained in either of the two analyses, while Specific EcoPeaks were defined when an FDR of 1% was obtained in both analyses (for more details, consult Roberts Kingman et al. 2021). Since the number of DSGs in our dataset was not very high, we used the Sensitive EcoPeaks to increase the power of our analyses. We tested the enrichment of our DEGs and DSGs in both Pacific and Global EcoPeaks. Since the EcoPeaks were originally identified in the v4.1 (*gasAcu1-4*) genome assembly (Roberts Kingman et al. 2021), we lifted over the coordinates of the EcoPeaks to the coordinates of the version 5 genome (supplementary table S6, Supplementary Material online) using the *liftOver()* function of the *R* package *rtracklayer* v1.46.0 (Lawrence et al. 2009) and the chain file “v4.1_to_v5.chain” available at the Stickleback Genome Browser (https://stickleback.genetics.uga.edu/).

Briefly, the enrichment analysis involved: (i) identifying the proportion of DEGs and DSGs that overlapped with EcoPeaks; (ii) identifying the proportion of background genes that overlapped with EcoPeaks; and (iii) testing if the proportion of DEGs and DSGs in EcoPeaks is significantly higher than the proportion of background genes using a permutation test (1,000 permutations) in R. The background genes included in this analysis corresponded to the genes that were tested for differential expression or differential splicing, respectively, in each dataset. For the differential expression analysis, the background genes are all that passed the minimum gene expression filter. For the differential splicing analysis, the background genes are limited to those genes for which rMATs found evidence of alternative splicing and were therefore tested for differential splicing between the marine and freshwater ecotypes. For the enrichment analysis of genes that were both differentially expressed and differentially spliced (DESGs), we used the intersection of the DEG and DSG background genes. Next, we asked whether certain types of DS events were more enriched in EcoPeaks than others. For each category of DS events, we assessed whether DSGs with that specific type of splicing event were more likely to overlap with EcoPeaks compared to background genes. Background genes were defined as all genes with that type of splicing event, regardless of whether they were differentially spliced between ecotypes.

We also tested whether DEGs and DSGs in EcoPeaks had stronger differential expression or splicing than genes outside EcoPeaks. We used a permutation test (10,000 permutations) to compare whether the median of the distribution of log2 fold-change of DEGs or the distribution of total isoform difference of all the DS events per DSG (i.e. the summed absolute isoform difference of all DS events in that DSG) was different for genes inside and outside of EcoPeaks. We also performed this test between different types of DS events. For each event type, we calculated the distribution of isoform differences of DS events of that type within and outside EcoPeaks and compared the distributions using a permutation test (10,000 permutations).

To test whether the DEGs and DSGs we identified might be involved in phenotypic divergence between the ecotypes, we did a similar enrichment analysis for QTL that underlie traits that differ between Pacific marine and freshwater populations (Peichel and Marques 2017; Rennison and Peichel 2022). We used the dataset of Liu et al. (2022), which excluded QTL that did not go in the expected direction (i.e. QTL with marine alleles that lead to more freshwater-like phenotypes and vice versa) (Liu et al. 2022). We lifted the coordinates of the QTL windows from v1 to v5 of the stickleback genome using the chain file “v1_withChrUn_to_v5.chain.txt” available at the Stickleback Genome Browser (https://stickleback.genetics.uga.edu/). Similar to the EcoPeak enrichment analysis, we: (i) identified the proportion of DEGs and DSGs that overlapped with QTL; (ii) identified the proportion of background genes that overlapped with QTL; and (iii) tested if the proportion of DEGs and DSGs in QTL is significantly higher than the proportion of background genes using a permutation test (1,000 permutations) in R. We did this for the complete set of QTL and also for the different phenotypic categories of QTL defined by Peichel and Marques (2017): defense, behavior and sensory system, body shape, body size, swimming, feeding, pigmentation, and respiration (supplementary table S4, Supplementary Material online). We did not test QTL category enrichment for the different types of DS events because we did not have enough DS events and DSGs for the analysis to have statistical power.

### Effect of Genetic Distance in Expression and Splicing

To complement the EcoPeak enrichment analysis, we also looked for evidence of transcriptome-wide selection for stronger differential expression (i.e. a higher log2 fold-change) and splicing (i.e. a greater isoform difference) by looking at the correlation of gene expression fold-change or splicing isoform difference with genetic divergence between ecotypes. A significant positive correlation could mean transcriptome-wide selection for stronger differential expression (DE) and differential splicing (DS). To obtain a measure of genetic distance in both coding and noncoding regions across the stickleback genome, we used the SNP-level *P*-value data used to identify Pacific EcoPeaks by Roberts Kingman et al. (2021) (data kindly provided by the authors). These *P*-values result from a Fisher Exact Test for the probability of an imbalance in allele counts between ecotypes at each SNP (for more details, consult Section S9 in Roberts Kingman et al. 2021). A *P*-value of <0.05 means that the SNP differs in allele frequencies between ecotypes and suggests divergent selection. As for the EcoPeak analysis, we translated all SNP coordinates from the v4.1 (*gasAcu1-4*) genome to the v5 coordinates. Then, for each gene in the transcriptome, we calculated a gene-level genetic divergence based on the average *P*-value of the SNPs overlapping each gene. With this data, we tested if there was a correlation between genetic distance and strength of differential expression or splicing using all the genes tested in the differential expression and differential splicing analyses, separately. In the case of genes with more than one alternative splicing event, the isoform difference of the AS or DS event with the highest isoform difference between ecotypes was selected. To test the significance of the correlations, we used a nonparametric linear model based on ranks implemented in the *R* package *Rfit* v0.24.2 (Kloke and McKean 2012). We also did this analysis separately for genes inside and outside of Pacific EcoPeaks to test whether EcoPeaks affected the relationship between genetic distance and gene expression or splicing.

### Comparisons Between Types of DS Events

To ask whether selection could be acting differently on the different types of splicing events, we compared both their genetic distance distributions and isoform difference distributions. For the isoform difference comparison, we calculated the total isoform difference of all DS events of the same type per DSG and compared the median of the average isoform difference distribution between the five types of splicing events. To compare the genetic difference of different DS types, we only included the DS event with the highest absolute isoform difference per DSG in order to avoid pseudo-replication of the same genes. We then compared the average SNP *P*-value of the DSGs with different types of DS event. For both analyses, we tested the pairwise difference of the medians of the distributions of each type of DS event using a Mann–Whitney *U* test, as implemented in the function wilcox.test() from the *R* package *stats* v3.6.1. We complemented this with a third analysis, in which we asked if DSGs had a higher genetic distance than genes with AS events, but no DS events between ecotypes. For each type of splicing event, we compared the median of the genetic distance distributions of the DSGs and the non-DSGs (genes with that type of AS event but no DS event). We tested the significance of the differences with a permutation test (10,000 permutations).

### Gene Ontology Enrichment Analysis

We tested whether DSGs and DEGs were enriched in specific biological processes, through a Gene Ontology Enrichment analysis. We used the g:Profiler (Reimand et al. 2007) as implemented in the *R* package *gprofiler2* v0.2.2. We used the *G. aculeatus* functional annotations from Ensembl implemented in *gprofiler* and used custom backgrounds for the statistical analysis. As for the EcoPeak and QTL enrichment analysis, the background for the DEGs was the set of genes that passed the minimum expression, and the background for the DSGs was the set of genes with evidence of AS that passed the minimum expression filter junction filtering process (i.e. the set of genes that were tested for DE by EdgeR and DS by rMATs). In addition, we also tested the enrichment of the subset of DEGs and DSGs that overlapped both Pacific EcoPeaks and QTL. Finally, we manually looked at the gene function of DSGs that overlapped both Global EcoPeaks and QTL in the GeneCards database (https://www.genecards.org/), which integrates information from several databases including NCBI Gene (https://www.ncbi.nlm.nih.gov/gene) and UniProt (https://www.uniprot.org/).

## Supplementary Material

evaf105_Supplementary_Data

## Data Availability

All RNA-seq data used in this study was already publicly available at NCBI's Sequence Read Archive (SRA) under BioProjects PRJNA371616 and PRJNA530695. Accession numbers for all samples used can be found in supplementary table S1, Supplementary Material online. The EcoPeak data can be obtained from the UCSC Genome Browser (http://genome.ucsc.edu/) (instructions for downloading the data are in Roberts Kingman et al. (2021), under the “Data and materials availability” section). QTL data can be found in the Supplementary material of Rennison and Peichel (2022) and Liu et al. (2022). Scripts used for the analyses are available online at the following GitHub repository: https://github.com/cryez/RodriguezRamirez-and-Peichel-2025.

## References

[evaf105-B1] Barroso GV, Puzovic N, Dutheil JY. The evolution of gene-specific transcriptional noise is driven by selection at the pathway level. Genetics. 2018:208(1):173–189. 10.1534/genetics.117.300467. 29097405 PMC5753856

[evaf105-B2] Bell MA, Foster SA. The evolutionary biology of the threespine stickleback. Oxford: Oxford University Press; 1994.

[evaf105-B3] Bolger AM, Lohse M, Usadel B. Trimmomatic: a flexible trimmer for Illumina sequence data. Bioinformatics. 2014:30(15):2114–2120. 10.1093/bioinformatics/btu170. 24695404 PMC4103590

[evaf105-B4] Bomblies K, Peichel CL. Genetics of adaptation. Proc Natl Acad Sci U S A. 2022:119(30):e2122152119. 10.1073/pnas.2122152119. 35858399 PMC9335183

[evaf105-B5] Bush SJ, Chen L, Tovar-Corona JM, Urrutia AO. Alternative splicing and the evolution of phenotypic novelty. Philos Trans R Soc Lond B Biol Sci. 2017:372(1713):20150474. 10.1098/rstb.2015.0474. 27994117 PMC5182408

[evaf105-B6] Carroll SB . Evolution at two levels: on genes and form. PLoS Biol. 2005:3(7):e245. 10.1371/journal.pbio.0030245. 16000021 PMC1174822

[evaf105-B7] Chan YF, Marks ME, Jones FC, Villarreal G, Shapiro MD, Brady SD, Southwick AM, Absher DM, Grimwood J, Schmutz J, et al Adaptive evolution of pelvic reduction in sticklebacks by recurrent deletion of a *Pitx1* enhancer. Science. 2010:327(5963):302–305. 10.1126/science.1182213. 20007865 PMC3109066

[evaf105-B8] Chen L, Bush SJ, Tovar-Corona JM, Castillo-Morales A, Urrutia AO. Correcting for differential transcript coverage reveals a strong relationship between alternative splicing and organism complexity. Mol Biol Evol. 2014:31(6):1402–1413. 10.1093/molbev/msu083. 24682283 PMC4032128

[evaf105-B9] Chen Y, McCarthy D, Ritchie M, Robinson M, Smyth G. 2008. edgeR: differential analysis of sequence read count data—User's Guide. https://www.bioconductor.org/packages/devel/bioc/vignettes/edgeR/inst/doc/edgeRUsersGuide.pdf.

[evaf105-B10] Colosimo PF, Hosemann KE, Balabhadra S, Villarreal G, Dickson M, Grimwood J, Schmutz J, Myers RM, Schluter D, Kingsley DM. Widespread parallel evolution in sticklebacks by repeated fixation of Ectodysplasin alleles. Science. 2005:307(5717):1928–1933. 10.1126/science.1107239. 15790847

[evaf105-B11] Courtier-Orgogozo V, Arnoult L, Prigent SR, Wiltgen S, Martin A. Gephebase, a database of genotype–phenotype relationships for natural and domesticated variation in Eukaryotes. Nucleic Acids Res. 2020:48(D1):D696–D703. 10.1093/nar/gkz796. 31544935 PMC6943045

[evaf105-B12] Ewels P, Magnusson M, Lundin S, Käller M. MultiQC: summarize analysis results for multiple tools and samples in a single report. Bioinformatics. 2016:32(19):3047–3048. 10.1093/bioinformatics/btw354. 27312411 PMC5039924

[evaf105-B13] Gibbons TC, Metzger DCH, Healy TM, Schulte PM. Gene expression plasticity in response to salinity acclimation in threespine stickleback ecotypes from different salinity habitats. Mol Ecol. 2017:26(10):2711–2725. 10.1111/mec.14065. 28214359

[evaf105-B14] Gracheva EO, Cordero-Morales JF, González-Carcacía JA, Ingolia NT, Manno C, Aranguren CI, Weissman JS, Julius D. Ganglion-specific splicing of TRPV1 underlies infrared sensation in vampire bats. Nature. 2011:476(7358):88–91. 10.1038/nature10245. 21814281 PMC3535012

[evaf105-B15] Grantham ME, Brisson JA. Extensive differential splicing underlies phenotypically plastic aphid morphs. Mol Biol Evol. 2018:35(8):1934–1946. 10.1093/molbev/msy095. 29722880 PMC6063273

[evaf105-B16] Hill MS, Vande Zande P, Wittkopp PJ. Molecular and evolutionary processes generating variation in gene expression. Nat Rev Genet. 2021:22(4):203–215. 10.1038/s41576-020-00304-w. 33268840 PMC7981258

[evaf105-B17] Hillmer EJ, Zhang H, Li HS, Watowich SS. STAT3 signaling in immunity. Cytokine Growth Factor Rev. 2016:31:1–15. 10.1016/j.cytogfr.2016.05.001. 27185365 PMC5050093

[evaf105-B18] Hoekstra HE, Coyne JA. The locus of evolution: evo devo and the genetics of adaptation. Evolution. 2007:61(5):995–1016. 10.1111/j.1558-5646.2007.00105.x. 17492956

[evaf105-B19] Howes TR, Summers BR, Kingsley DM. Dorsal spine evolution in threespine sticklebacks via a splicing change in MSX2A. BMC Biol. 2017:15(1):115. 10.1186/s12915-017-0456-5. 29212540 PMC5719529

[evaf105-B20] Indjeian VB, Kingman GA, Jones FC, Guenther CA, Grimwood J, Schmutz J, Myers RM, Kingsley DM. Evolving new skeletal traits by *cis*-regulatory changes in bone morphogenetic proteins. Cell. 2016:164(1-2):45–56. 10.1016/j.cell.2015.12.007. 26774823 PMC4759241

[evaf105-B21] Ishikawa A, Kabeya N, Ikeya K, Kakioka R, Cech JN, Osada N, Leal MC, Inoue J, Kume M, Toyoda A, et al A key metabolic gene for recurrent freshwater colonization and radiation in fishes. Science. 2019:364(6443):886–889. 10.1126/science.aau5656. 31147520

[evaf105-B22] Jacobs A, Elmer KR. Alternative splicing and gene expression play contrasting roles in the parallel phenotypic evolution of a salmonid fish. Mol Ecol. 2021:30(20):4955–4969. 10.1111/mec.15817. 33502030 PMC8653899

[evaf105-B23] Jones FC, Grabherr MG, Chan YF, Russell P, Mauceli E, Johnson J, Swofford R, Pirun M, Zody MC, White S, et al The genomic basis of adaptive evolution in threespine sticklebacks. Nature. 2012:484(7392):55–61. 10.1038/nature10944. 22481358 PMC3322419

[evaf105-B24] King MC, Wilson AC. Evolution at two levels in humans and chimpanzees. Science. 1975:188(4184):107–116. 10.1126/science.1090005. 1090005

[evaf105-B25] Kloke JD, McKean JW. Rfit: rank-based estimation for linear models. R J. 2012:4(2):57–64. 10.32614/RJ-2012-014.

[evaf105-B26] Kondrashov FA . Origin of alternative splicing by tandem exon duplication. Hum Mol Genet. 2001:10(23):2661–2669. 10.1093/hmg/10.23.2661. 11726553

[evaf105-B27] Lawrence M, Gentleman R, Carey V. rtracklayer: an R package for interfacing with genome browsers. Bioinformatics. 2009:25(14):1841–1842. 10.1093/bioinformatics/btp328. 19468054 PMC2705236

[evaf105-B28] Letunic I . Common exon duplication in animals and its role in alternative splicing. Hum Mol Genet. 2002:11(13):1561–1567. 10.1093/hmg/11.13.1561. 12045209

[evaf105-B29] Lev-Maor G, Goren A, Sela N, Kim E, Keren H, Doron-Faigenboim A, Leibman-Barak S, Pupko T, Ast G. The “alternative” choice of constitutive exons throughout evolution. PLoS Genet. 2007:3(11):e203. 10.1371/journal.pgen.0030203. 18020709 PMC2077895

[evaf105-B30] Levy DE, Lee C. What does Stat3 do? J Clin Invest. 2002:109(9):1143–1148. 10.1172/JCI0215650. 11994402 PMC150972

[evaf105-B31] Liao Y, Smyth GK, Shi W. featureCounts: an efficient general purpose program for assigning sequence reads to genomic features. Bioinformatics. 2014:30(7):923–930. 10.1093/bioinformatics/btt656. 24227677

[evaf105-B32] Lin L, Jiang P, Park JW, Wang J, Lu Z, Lam MPY, Ping P, Xing Y. The contribution of Alu exons to the human proteome. Genome Biol. 2016:17:15. 10.1186/s13059-016-0876-5. 26821878 PMC4731929

[evaf105-B33] Liu Z, Roesti M, Marques D, Hiltbrunner M, Saladin V, Peichel CL. Chromosomal fusions facilitate adaptation to divergent environments in threespine stickleback. Mol Biol Evol. 2022:39(2):msab358. 10.1093/molbev/msab358. 34908155 PMC8826639

[evaf105-B34] Manahan DN, Nachman MW. Alternative splicing and environmental adaptation in wild house mice. Heredity (Edinb). 2024:132(3):133–141. 10.1038/s41437-023-00663-0. 38012302 PMC10923775

[evaf105-B35] Maritano D, Sugrue ML, Tininini S, Dewilde S, Strobl B, Fu X, Murray-Tait V, Chiarle R, Poli V. The STAT3 isoforms α and β have unique and specific functions. Nat Immunol. 2004:5(4):401–409. 10.1038/ni1052. 15021879

[evaf105-B36] Martin A, Orgogozo V. The loci of repeated evolution: a catalog of genetic hotspots of phenotypic variation. Evolution. 2013:67(5):1235–1n/a. 10.1111/evo.12081. 23617905

[evaf105-B37] Martinez-Gomez L, Abascal F, Jungreis I, Pozo F, Kellis M, Mudge JM, Tress ML. Few SINEs of life: Alu elements have little evidence for biological relevance despite elevated translation. NAR Genom Bioinform. 2020:2(1):lqz023. 10.1093/nargab/lqz023. 31886458 PMC6924539

[evaf105-B38] Miller CT, Beleza S, Pollen AA, Schluter D, Kittles RA, Shriver MD, Kingsley DM. *cis*-Regulatory changes in Kit ligand expression and parallel evolution of pigmentation in sticklebacks and humans. Cell. 2007:131(6):1179–1189. 10.1016/j.cell.2007.10.055. 18083106 PMC2900316

[evaf105-B39] Nath S, Shaw DE, White MA. Improved contiguity of the threespine stickleback genome using long-read sequencing. G3 (Bethesda, Md.). 2021:11(2):jkab007. 10.1093/g3journal/jkab007. 33598708 PMC8022941

[evaf105-B40] O'Shea JJ, Gadina M, Schreiber RD. Cytokine signaling in 2002. Cell. 2002:109(2):S121–S131. 10.1016/S0092-8674(02)00701-8. 11983158

[evaf105-B41] Peichel CL, Marques DA. The genetic and molecular architecture of phenotypic diversity in sticklebacks. Philos Trans R Soc Lond B Biol Sci. 2017:372(1713):20150486. 10.1098/rstb.2015.0486. 27994127 PMC5182418

[evaf105-B42] R Core Team . 2019. R: a language and environment for statistical computing [accessed 2023 Jan 19]. https://www.R-project.org/.

[evaf105-B43] Rebeiz M, Pool JE, Kassner VA, Aquadro CF, Carroll SB. Stepwise modification of a modular enhancer underlies adaptation in a *Drosophila* population. Science. 2009:326(5960):1663–1667. 10.1126/science.1178357. 20019281 PMC3363996

[evaf105-B44] Reimand J, Kull M, Peterson H, Hansen J, Vilo J. g:Profiler—a web-based toolset for functional profiling of gene lists from large-scale experiments. Nucleic Acids Res. 2007:35(suppl_2):W193–W200. 10.1093/nar/gkm226. 17478515 PMC1933153

[evaf105-B45] Rennison DJ, Peichel CL. Pleiotropy facilitates parallel adaptation in sticklebacks. Mol Ecol. 2022:31(5):1476–1486. 10.1111/mec.16335. 34997980 PMC9306781

[evaf105-B46] Richard M, Yvert G. How does evolution tune biological noise? Front Genet. 2014:5:374. 10.3389/fgene.2014.00374. 25389435 PMC4211553

[evaf105-B47] Ritchie ME, Phipson B, Wu D, Hu Y, Law CW, Shi W, Smyth G. limma powers differential expression analyses for RNA-sequencing and microarray studies. Nucleic Acids Res. 2015:43(7):e47. 10.1093/nar/gkv007. 25605792 PMC4402510

[evaf105-B48] Roberts Kingman GA, Vyas DN, Jones FC, Brady SD, Chen HI, Reid K, Milhaven M, Bertino TS, Aguirre WE, Heins DC, et al Predicting future from past: the genomic basis of recurrent and rapid stickleback evolution. Sci Adv. 2021:7(25):eabg5285. 10.1126/sciadv.abg5285. 34144992 PMC8213234

[evaf105-B49] Rodríguez-Ramírez CE, Hiltbrunner M, Saladin V, Walker S, Urrutia A, Peichel CL. Molecular mechanisms of *Eda*-mediated adaptation to freshwater in threespine stickleback. Mol Ecol. 2023. 10.1111/mec.16989. PMC12288785 37194086

[evaf105-B50] Rogers TF, Palmer DH, Wright AE. Sex-specific selection drives the evolution of alternative splicing in birds. Mol Biol Evol. 2021:38(2):519–530. 10.1093/molbev/msaa242. 32977339 PMC7826194

[evaf105-B51] Shen S, Park JW, Lu Z, Lin L, Henry MD, Wu YN, Zhou Q, Xing Y. rMATS: robust and flexible detection of differential alternative splicing from replicate RNA-Seq data. Proc Natl Acad Sci U S A. 2014:111(51):e5593–e5601. 10.1073/pnas.1419161111. 25480548 PMC4280593

[evaf105-B52] Singh P, Ahi EP. The importance of alternative splicing in adaptive evolution. Mol Ecol. 2022:31(7):1928–1938. 10.1111/mec.16377. 35094439

[evaf105-B53] Singh P, Börger C, More H, Sturmbauer C. The role of alternative splicing and differential gene expression in cichlid adaptive radiation. Genome Biol Evol. 2017:9(10):2764–2781. 10.1093/gbe/evx204. 29036566 PMC5737861

[evaf105-B54] Smith CCR, Tittes S, Mendieta JP, Collier-zans E, Rowe HC, Rieseberg LH, Kane NC. Genetics of alternative splicing evolution during sunflower domestication. Proc Natl Acad Sci U S A. 2018:115(26):6768–6773. 10.1073/pnas.1803361115. 29891718 PMC6042098

[evaf105-B55] Sorek R . The birth of new exons: mechanisms and evolutionary consequences. RNA. 2007:13(10):1603–1608. 10.1261/rna.682507. 17709368 PMC1986822

[evaf105-B56] Sorek R, Ast G, Graur D. *Alu*-containing exons are alternatively spliced. Genome Res. 2002:12(7):1060–1067. 10.1101/gr.229302. 12097342 PMC186627

[evaf105-B57] Stern DL, Orgogozo V. The loci of evolution: how predictable is genetic evolution? Evolution. 2008:62(9):2155–2177. 10.1111/j.1558-5646.2008.00450.x. 18616572 PMC2613234

[evaf105-B58] Steward RA, de Jong MA, Oostra V, Wheat CW. Alternative splicing in seasonal plasticity and the potential for adaptation to environmental change. Nat Commun. 2022:13(1):755. 10.1038/s41467-022-28306-8. 35136048 PMC8825856

[evaf105-B59] Sun W, You X, Gogol-Döring A, He H, Kise Y, Sohn M, Chen T, Klebes A, Schmucker D, Chen W. Ultra-deep profiling of alternatively spliced *Drosophila* Dscam isoforms by circularization-assisted multi-segment sequencing. EMBO J. 2013:32(14):2029–2038. 10.1038/emboj.2013.144. 23792425 PMC3715862

[evaf105-B60] Tovar-Corona JM, Castillo-Morales A, Chen L, Olds BP, Clark JM, Reynolds SE, Pittendrigh BR, Feil EJ, Urrutia AO. Alternative splice in alternative lice. Mol Biol Evol. 2015:32(10):2749–2759. 10.1093/molbev/msv151. 26169943 PMC4576711

[evaf105-B61] Verta J-P, Jacobs A. The role of alternative splicing in adaptation and evolution. Trends Ecol Evol. 2022:37(4):299–308. 10.1016/j.tree.2021.11.010. 34920907

[evaf105-B62] Verta J-P, Jones FC. Predominance of *cis*-regulatory changes in parallel expression divergence of sticklebacks. Elife. 2019:8:e43785. 10.7554/eLife.43785. 31090544 PMC6550882

[evaf105-B63] Wang X, Yang M, Ren D, Terzaghi W, Deng X, He G. *Cis*-regulated alternative splicing divergence and its potential contribution to environmental responses in *Arabidopsis*. Plant J. 2019:97(3):555–570. 10.1111/tpj.14142. 30375060

[evaf105-B64] Wegrzyn J, Potla R, Chwae Y-J, Sepuri NBV, Zhang Q, Koeck T, Derecka M, Szczepanek K, Szelag M, Gornicka A, et al Function of mitochondrial Stat3 in cellular respiration. Science. 2009:323(5915):793–797. 10.1126/science.1164551. 19131594 PMC2758306

[evaf105-B65] Wittkopp PJ, Kalay G. *Cis*-regulatory elements: molecular mechanisms and evolutionary processes underlying divergence. Nat Rev Genet. 2012:13(1):59–69. 10.1038/nrg3095. 22143240

[evaf105-B66] Wong JJ-L, Au AYM, Ritchie W, Rasko JEJ. Intron retention in mRNA: no longer nonsense: known and putative roles of intron retention in normal and disease biology. BioEssays. 2016:38(1):41–49. 10.1002/bies.201500117. 26612485

[evaf105-B67] Wooldridge TB, Kautt AF, Lassance J-M, McFadden S, Domingues VS, Mallarino R, Hoekstra HE. An enhancer of *Agouti* contributes to parallel evolution of cryptically colored beach mice. Proc Natl Acad Sci U S A. 2022:119(27):e2202862119. 10.1073/pnas.2202862119. 35776547 PMC9271204

[evaf105-B68] Wright CJ, Smith CWJ, Jiggins CD. Alternative splicing as a source of phenotypic diversity. Nat Rev Genet. 2022:23(11):697–710. 10.1038/s41576-022-00514-4. 35821097

[evaf105-B69] Xia B, Zhang W, Zhao G, Zhang X, Bai J, Brosh R, Wudzinska A, Huang E, Ashe H, Ellis G, et al On the genetic basis of tail-loss evolution in humans and apes. Nature. 2024:626(8001):1042–1048. 10.1038/s41586-024-07095-8. 38418917 PMC10901737

[evaf105-B70] Xing Y, Lee C. Alternative splicing and RNA selection pressure—evolutionary consequences for eukaryotic genomes. Nat Rev Genet. 2006:7(7):499–509. 10.1038/nrg1896. 16770337

[evaf105-B71] Xiong S, Wang W, Kenzior A, Olsen L, Krishnan J, Persons J, Medley K, Peuß R, Wang Y, Chen S, et al Enhanced lipogenesis through Pparγ helps cavefish adapt to food scarcity. Curr Biol. 2022:32(10):2272–2280.e6. 10.1016/j.cub.2022.03.038. 35390280 PMC9133166

[evaf105-B72] Zhang Y, Parmigiani G, Johnson WE. ComBat-seq: batch effect adjustment for RNA-seq count data. NAR Genom Bioinform. 2020:2(3):lqaa078. 10.1093/nargab/lqaa078. 33015620 PMC7518324

[evaf105-B73] Zhou S, Dai Q, Huang X, Jin A, Yang Y, Gong X, Xu H, Gao X, Jiang L. STAT3 is critical for skeletal development and bone homeostasis by regulating osteogenesis. Nat Commun. 2021:12(1):6891. 10.1038/s41467-021-27273-w. 34824272 PMC8616950

