# Supplementary material for: The Role of Alternative Splicing in Marine–Freshwater Divergence in Threespine Stickleback
Source: Genome Biol Evol. 2025 May 28;17(6):evaf105. doi: 10.1093/gbe/evaf105 (PMC12159805; doi:10.1093/gbe/evaf105)
Supplement: evaf105_Supplementary_Data [file evaf105_supplementary_data.zip › SupplementaryFigures_gbe_3rd_round.pdf]

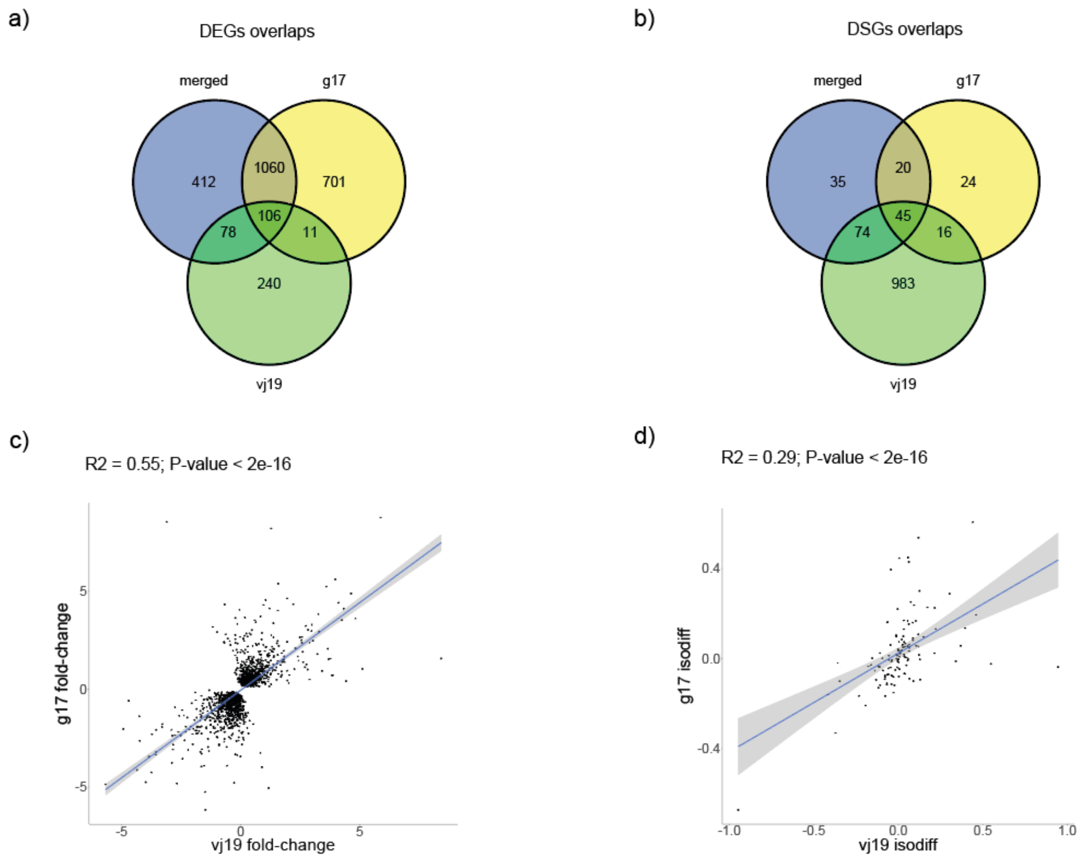

Supplementary Figure S1. Comparison between the samples from Gibbons et al. 2017 (g17) and Verta and Jones 2019 (vj19) used in this study. Venn diagrams represent the overlap of (a) DEGs and (b) DSGs among the individually analysed datasets and the datasets when they are analysed together (merged dataset). Correlation plots show the correlation of (c) expression fold-changes between the two datasets for genes that are DEGs in the merged dataset and (d) in isoform differences between the two datasets for genes that are DSGs in the merged dataset.

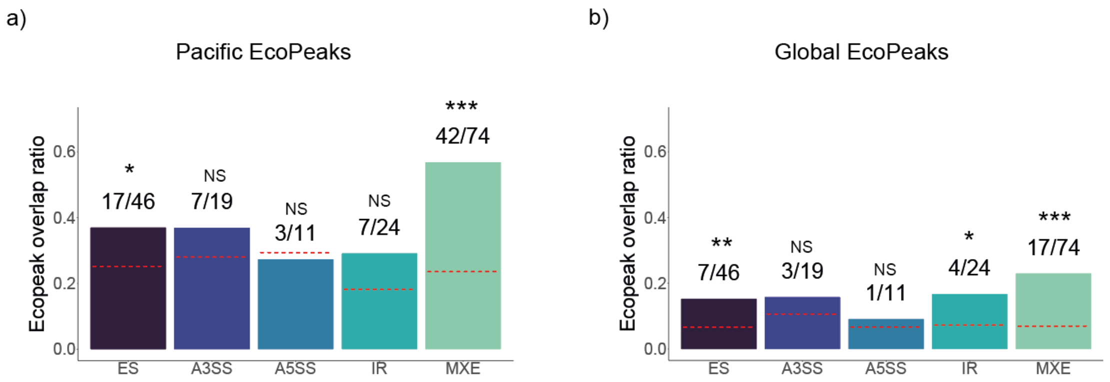

Supplementary Figure S2. Proportion of differential splicing (DS) events of each type found in genes within Pacific EcoPeaks (a) or Global EcoPeaks (b). Red dashed lines represent the proportion of genes in the transcriptome with that type of AS event (even if not DS) found inside EcoPeaks, which is the baseline against which enrichment of DS events in EcoPeaks was tested. The proportions of significant DS events inside the EcoPeaks versus the total number of DS events of that type are shown on top of each bar. Asterisks represent significance levels for the EcoPeak enrichment test (permutation test, 1000 permutations): *p-value < 0.05; **p-value < 0.01; ***p-value < 0.001; NS – not significant, p-value > 0.05.

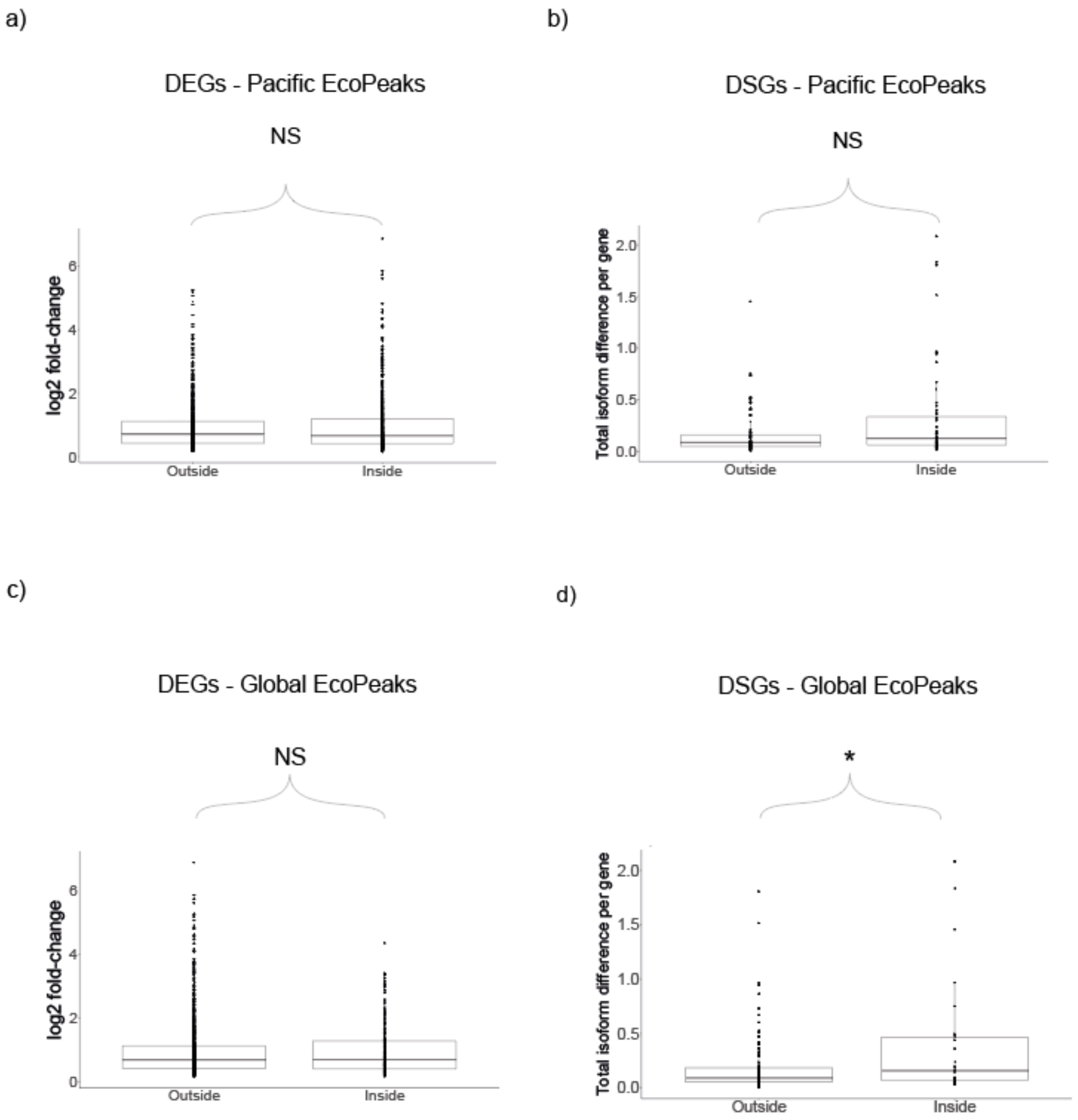

Supplementary Figure S3. Difference in fold-change of DEGs (a, c) and isoform difference of DSGs (b, d) inside and outside of Pacific (a, b) or Global (c, d) EcoPeaks. Asterisks represent significance levels for the difference of the medians (permutation test, 1000 permutations): *p-value < 0.05; NS – not significant, p-value > 0.05.

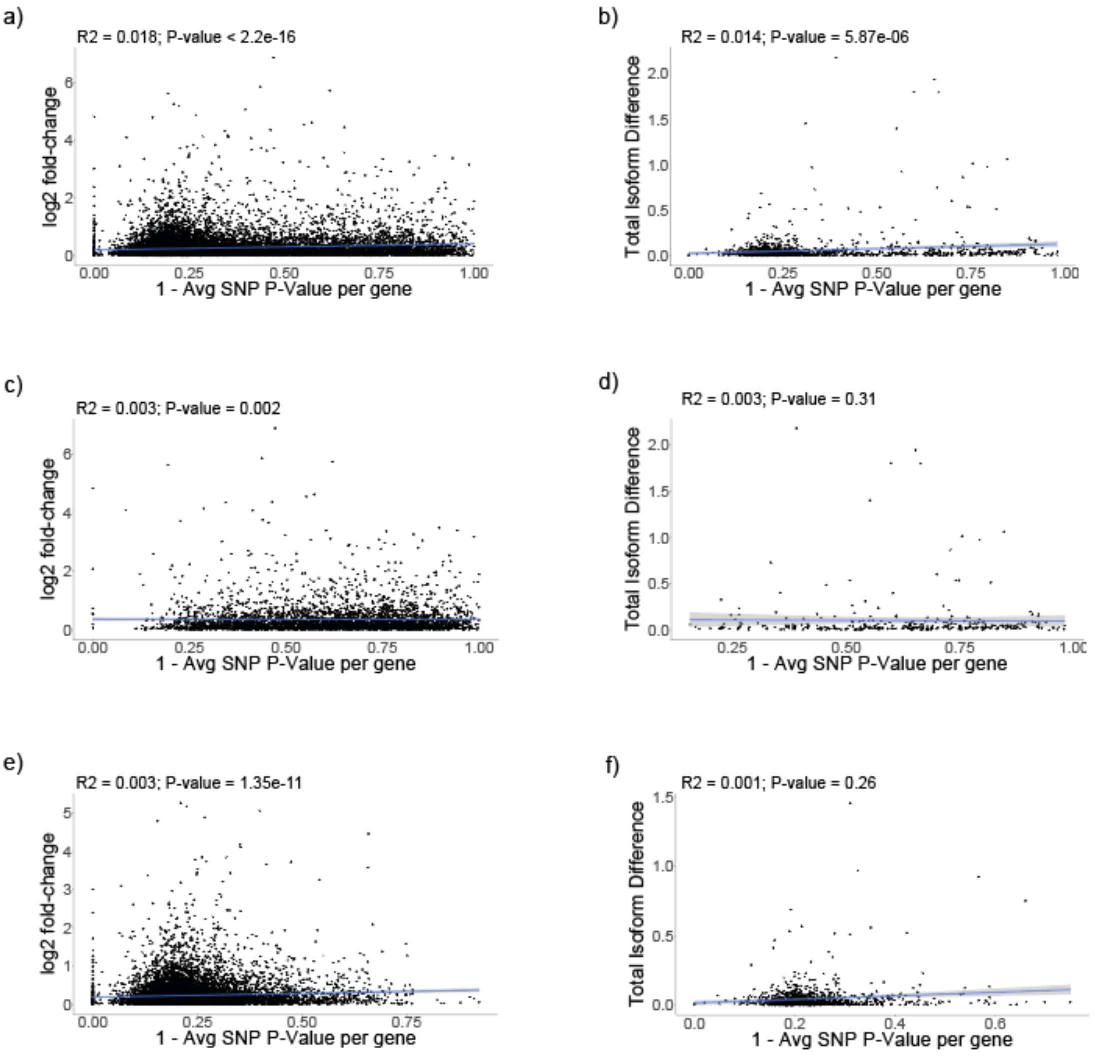

Supplementary Figure S4. Correlation between SNP average p-value and log2 fold-change (a, c, e) or isoform difference (b, d, f) for all genes tested for differential expression or splicing (a, b), only the genes inside Pacific EcoPeaks (c, d), or only the genes outside of Pacific EcoPeaks (e, f). The average SNP p-value is subtracted from 1 so that higher values on the x-axis represent higher genetic divergence of the SNPs in the genes.

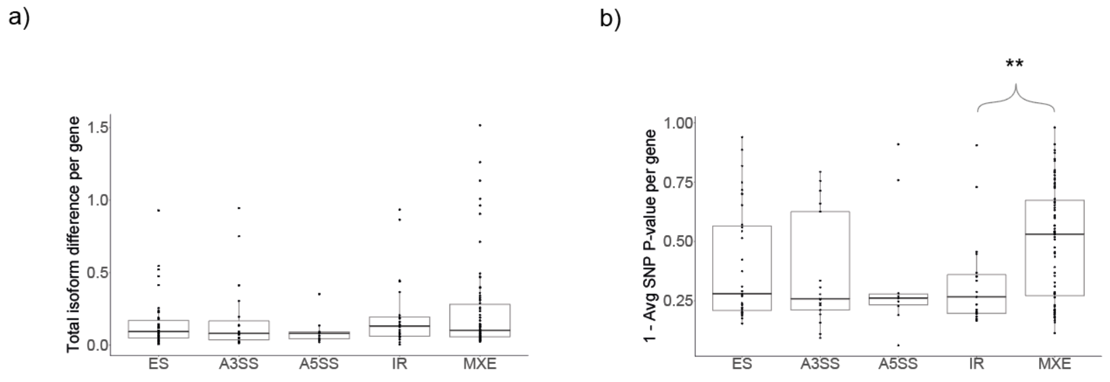

Supplementary Figure S5. Comparisons of the distribution of isoform differences (a) and SNP average p-value (b) between DSGs with different types of alternative splicing. Only the strongest splicing event per DSG was considered. In panel b, the average SNP p-value is subtracted from 1 so that larger values on the y-axis represent greater genetic divergence. Asterisks represent significant differences in the medians of the distributions according to a Mann Whitney U Test (p-value < 0.05). All pairwise combinations were tested, and only significant differences are shown.

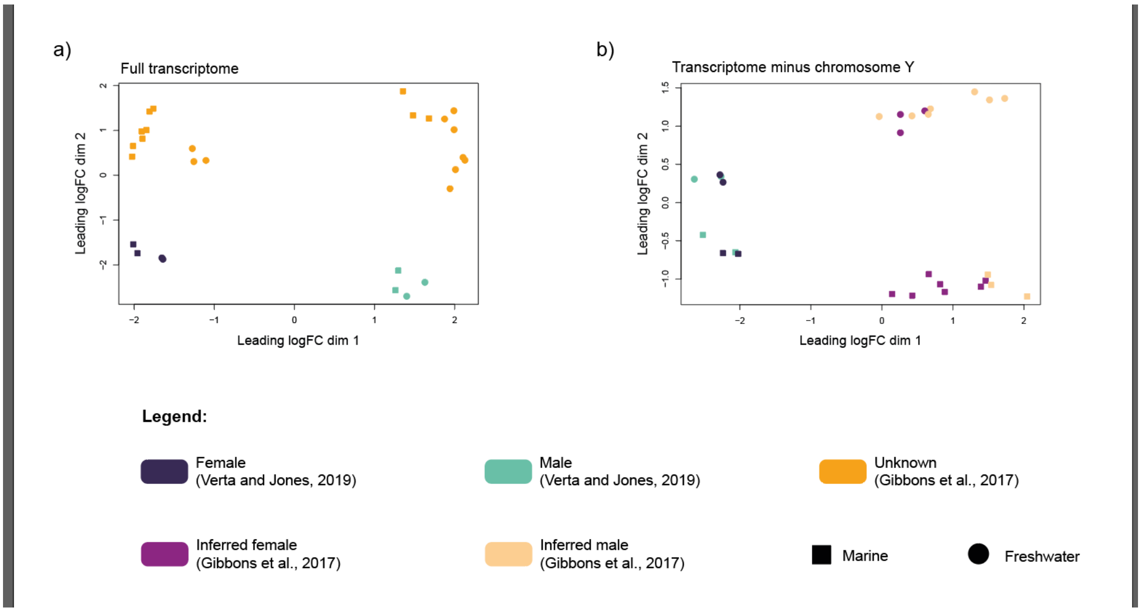

Supplementary Figure S6. Multidimensional scaling (MDS) plot of samples used in this study based on the top 500 genes with the largest pairwise distances between samples. These top 500 genes are drawn from (a) the full transcriptome including the Y chromosome or (b) the full transcriptome excluding the Y chromosome. In panel (b), the inferred sexes for the Gibbons et al. 2017 samples are assigned based on the MDS results in panel (a), where samples are clustered by sex in dimension 1.

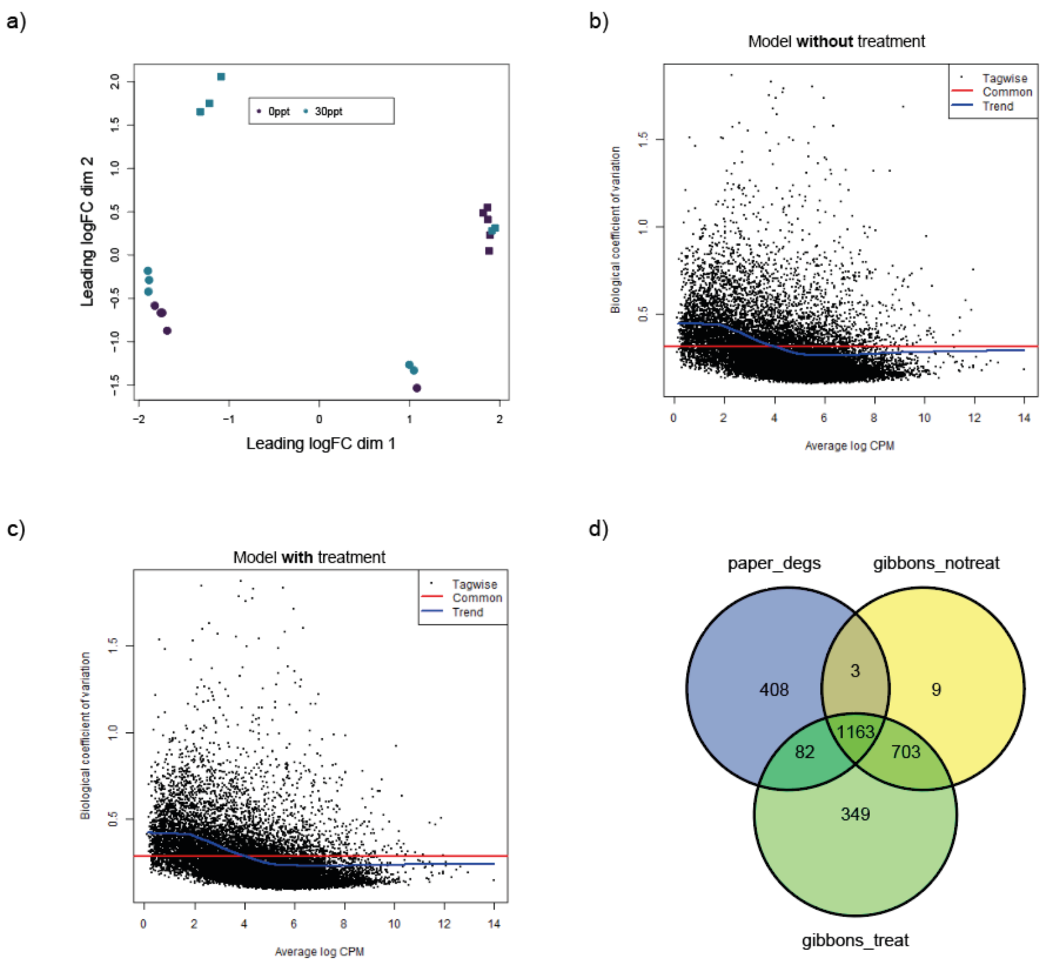

Supplementary Figure S7. Analyses of the effect of "treatment" in the Gibbons et al. (2017) dataset. There is no clustering by treatment in multidimensional scaling (MDS) plots (a), with samples from the 0 ppt salinity treatment in purple and samples from the 30 ppt salinity treatment in cyan. The first dimension separates male and female samples, and the second dimension separates samples by ecotype (freshwater samples indicated by circles, marine samples indicated by squares). Biological Coefficient of Variance (BCV) plots using a model without (b) and with (c) the treatment effect. The trend line (in blue) is slightly lower and thus there is slightly less variation when analyzing the data without the treatment effect than with the treatment effect. (d) Overlap between DEGs in the manuscript merged dataset (blue), DEGs in the Gibbons dataset without including treatment in the model (yellow), and DEGs in the Gibbons dataset when including treatment in the model (green).
